# Design Guidelines of Mobile Apps for Older Adults: Systematic Review and Thematic Analysis

**DOI:** 10.2196/43186

**Published:** 2023-09-21

**Authors:** Miguel Gomez-Hernandez, Xavier Ferre, Cristian Moral, Elena Villalba-Mora

**Affiliations:** 1 Center for Biomedical Technology Universidad Politécnica de Madrid Pozuelo de Alarcón Spain; 2 Emerging Technologies Research Lab Department of Human-Centred Computing Monash University Melbourne Australia; 3 School of Software Engineering Tongji University Shanghai China; 4 Biomedical Research Networking Center in Bioengineering Biomaterials and Nanomedicine (CIBER-BBN) Madrid Spain

**Keywords:** tablet, smartphone, older user, design recommendations, usability testing, user experience design, UX design, design, mobile app, tool, quality of life, software, training, visual design, older adults, mobile phone

## Abstract

**Background:**

Mobile apps are fundamental tools in today’s society for practical and social endeavors. However, these technologies are often not usable for older users. Given the increased use of mobile apps by this group of users and the impact that certain services may have on their quality of life, such as mobile health, personal finance, or online administrative procedures, a clear set of guidelines for mobile app designers is needed. Existing recommendations for older adults focus on investigations with certain groups of older adults or have not been extracted from experimental results.

**Objective:**

In this research work, we systematically reviewed the scientific literature that provided recommendations for the design of mobile apps based on usability testing with older adults and organized such recommendations into a meaningful set of design guidelines.

**Methods:**

We conducted a systematic literature review of journal and conference articles from 2010 to 2021. We included articles that carried out usability tests with populations aged >60 years and presented transferable guidelines on mobile software design, resulting in a final set of 40 articles. We then carried out a thematic analysis with 3 rounds of analysis to provide meaning to an otherwise diverse set of recommendations. At this stage, we discarded recommendations that were made by just 1 article, were based on a specific mobile app and were therefore nontransferrable, were based on other authors’ literature (as opposed to recommendations based on the results of usability tests), or were not sufficiently argued. With the remaining recommendations, we identified commonalities, wrote a faithful statement for each guideline, used a common language for the entire set, and organized the guidelines into categories, thereby giving shape to an otherwise diverse set of recommendations.

**Results:**

Among the 27 resulting guidelines, the rules *Simplify* and *Increase the size and distance between interactive controls* were transversal and of the greatest significance. The rest of the guidelines were divided into 5 categories (*Help & Training*, *Navigation*, *Visual Design*, *Cognitive Load*, and *Interaction*) and consequent subcategories in *Visual Design* (*Layout*, *Icons*, and *Appearance*) and *Interaction* (*Input* and *Output*). The recommendations were structured, explained in detail, and illustrated with applied examples extracted from the selected studies, where appropriate. We discussed the design implications of applying these guidelines, contextualized with relevant studies. We also discussed the limitations of the approach followed, stressing the need for further experimentation to gain a better understanding of how older adults use mobile apps and how to better design such apps with these users in mind.

**Conclusions:**

The compiled guidelines support the design of mobile apps that cater to the needs of older adults because they are based on the results of actual usability tests with users aged >60 years.

## Introduction

### Background

Mobile apps are becoming increasingly prevalent in the lives of older adults. The Pew Internet Research Center reported that 42% of older Americans (aged >65 years) had a smartphone and 32% owned a tablet in 2017, compared with 18% and 27% in 2013, respectively [1,2]. The importance of mobile apps for older adults became more apparent during recent events such as the COVID-19 pandemic to mitigate the effects of undesired self-isolation. Simultaneously, the older population is growing globally [3]. Because the interplay between mobile apps and older populations is gaining relevance, this paper pays special attention to both.

Touchscreen interfaces allow intuitive and direct manipulation interactions that depict real-world metaphors [4]. However, mobile devices still present substantial difficulties for older people with their nonconventional input methods and the limited size of their displays [5,6]. Other potential challenges include unexpected sensitivity of the touch surface, nonintuitive multifinger gestures, and a conceptual model that differs from desktop computers [4]. Knowledge about recommendations to design mobile apps tailored to address the limitations that older users experience can lead to a better adoption of mobile technologies by this population.

Some of the reasons older adults use mobile apps are to remain independent and active in society [7], monitor and improve their health condition through mobile health (mHealth) [8-10], or remember important information [11]. However, when using technology, older people encounter physical (visual, auditory, and motor changes and dexterity) and cognitive (decline in memory and attention) disadvantages associated with the aging process [12]. In relation to visual perception, declines in contrast sensitivity, acuity, and the ability to discriminate colors can affect symbol and character identification, button-striking accuracy, and reading rates [13]. The lack of motivation, experience, knowledge, access, understanding, and usability also challenge the adoption of technologies [14]. Therefore, mobile design efforts must address the needs and expectations of older adults.

The design of mobile apps needs to consider the user experience (UX) of older users and base design decisions on the results of usability tests with this group of users. The International Organization for Standardization defines usability as *“*the extent to which a product can be used by specified users to achieve specified goals with effectiveness, efficiency, and satisfaction in a specified context of use*”* [15]. However, the design of mobile apps for older adults has neglected some usability aspects. Design guidelines have focused extensively on visual and haptic issues (eg, high contrast, button type, and button size), whereas textual interface elements have been disregarded (eg, ease of text entry, button feedback, and font type) [16]. Therefore, this paper aimed to analyze all aspects of mobile app interaction design based on usability testing results.

When dealing with usability, we need to consider the overall design approach beyond user interface (UI) design, taking a user-centered design focus. The significance of involving older users in the design of mobile apps must be recognized if they constitute all or part of the target user population [17]. In addition to involving older adults (ie, end users) in testing the usability of mobile apps, user-centered evaluation can include experts [18]. Petrovčič et al [16] pointed out that good evaluations include both end users and experts. Dickinson et al [19] discussed that the challenge of including older users in technology design is that they demand additional technical, organizational, and managerial resources, compared with evaluations with experts. For example, one of the difficulties found in the segment of older people is that they are a heterogeneous group that modifies, uses, and interacts with technology in diverse ways [20].

Designing for older people is not a new realm. Within the human-computer interaction (HCI) field, interest in the topic of aging in connection with technology has grown, as have research-derived guidelines for the design of mobile apps that target older adults. However, these guidelines can be confusing, contradictory, complex, or have become obsolete [21]. Nurgalieva et al [21] and Petrovčič et al [16] also pointed out the limited number of validations, repeatability, and reproducibility of guideline-related studies. Therefore, this research work attempts to extract usability guidelines that are based on experimentation with older adults.

When referring to older adults, different authors have used different definitions. The definition of older adults is context dependent, and the generalizability of the findings for older people may distort the characteristics of this population. Vines et al [22] adopted a critical approach to the existing research that presented older adults as a homogeneous group. Older adults from technologically advanced countries might not experience mobile apps similar to older adults from regions where technology is less prevalent. There is also a difference in the use of the technology by those aged 60-74 years versus those aged >85 years. In this paper, older adults were defined as those aged ≥60 years.

### Objective

In summary, we aimed to create a set of design guidelines for mobile apps for older adults that stemmed from published usability testing results with these user cohorts. Thus, our research question was as follows: *What are the demonstrated heuristics to carry out the design of mobile apps for older adults?* We described similar studies; the methodology used; and the results obtained, in particular, the proposed set of design recommendations extracted from the selected studies and discussed the results in the context of related literature.

### Previous Work

Numerous mobile app design guidelines for older people have been developed through end-user evaluations (such as those included in this review) and heuristic evaluations [5,6,23]. Others have reviewed some of the literature (not systematically) on the design of mobile apps to propose a few guidelines [24-27]. In addition, Iancu and Iancu [28] provided a theoretical overview of the subject.

Some scoping reviews of the literature analyzed mHealth solutions for older adults [8,9,29,30]. Nimmanterdwong et al [31] reviewed the literature to illustrate the challenges and opportunities of applying human-centered design methodologies in the creation of mHealth solutions for older adults. Furthermore, Nurgalieva et al [21] systematically reviewed the trends and gaps in touchscreen design guidelines for older adults to systematize their knowledge of abilities and design categories. They focused on the characteristics of the older population, the quality of methods, and efforts to catalog the guidelines. In their review, they included studies grounded in secondary data (eg, literature reviews) and expert evaluations, which were excluded in our review. Another difference with our review stems from their definition of older adults (people aged >55 years), and their inclusion of guidelines specifically centered on certain pathologies (eg, Alzheimer disease). Petrovčič et al [16] also presented a systematic review of guidelines from 9 expert evaluations. Other systematic reviews on mobile apps for older users focused on specific health conditions such as cognitive decline [32] or older users’ cognitive, visual, and psychomotor challenges with mobile apps [33].

To our knowledge, no systematic review has extracted recommendations for mobile app design from primary data collected through evaluation activities, such as usability tests with older adults aged >60 years.

## Methods

### Overview

We performed a 2-step study starting with a systematic review and subsequently conducted a thematic analysis. We followed the PRISMA (Preferred Reporting Items for Systematic Reviews and Meta-Analyses) statement [34] recommendations for conducting systematic literature reviews. The following subsections detail the search strategies, eligibility criteria, data extraction, quality assessment, and analysis methods. For the review, we collected and evaluated the publications collaboratively using Parsifal, an online tool that supports the performance of systematic reviews. We chose the systematic literature review method because it offers a comprehensive and clear overview of the guidelines written to date and identifies gaps and further research topics.

### Search Strategy

First, we derived search terms from our research question: “older people,” “mobile,” and “usability.” The term “guideline” was not considered for 3 reasons: our endeavor was to unpack guidelines based on primary data resulting from usability evaluations; oftentimes, the term “guideline” is not used explicitly; and sometimes, recommendations are offered implicitly throughout the article. We selected the most relevant databases in the field of this review (Web of Science, IEEE, and Scopus) to broadly cover the literature on usability studies that address aging and mobile technology. Then, we tested different strings in the 3 databases to identify various spellings and synonyms. The final search string was as follows: “(older* OR elder* OR ageing OR aging OR senior*) AND (mobile* OR smartphone* OR tablet*) AND (usability* OR UX OR ‘’user experience’’ OR acceptability OR acceptance).”

To keep our search broad, we applied our search string to the fields of “title,” “abstract,” and “keywords” in each database. We searched for conference papers and academic articles written in English and published in peer-reviewed scientific journals and proceedings within the last 12 years (from 2010 to early 2021). The search strategy was discussed by all authors and conducted by the first author. The initial search yielded 4168 articles.

### Eligibility Criteria

The second step of the process consisted of refining the search by filtering the previous results to retain only the articles that met our eligibility criteria. First, we discarded duplicate articles (n=889). Then, we applied our inclusion and exclusion criteria to the remaining articles and filtered them by title, abstract, and keywords. The first inclusion or exclusion of articles was carried out by the first author who held weekly meetings with the rest of the authors to specify which exclusion criteria were applied and to check how the review was progressing. The exclusion criteria are shown in Textbox 1.

**Exclusion criteria.**

- Articles that were not written in English.
- Articles that were ≤4 pages long. In our experience, articles shorter than 4 pages usually did not have robust discussions and details that support the design recommendations offered.
- Articles not dealing with mobile apps. We defined mobile apps as those that run on tablets, smartphones, or smartwatches.
- Articles without an explicit usability test. We excluded qualitative research articles, heuristic evaluations, surveys of attitudes, expert reviews, and literature reviews that did not perform a usability test with older adults (aged >60 years). We considered a usability test to be a test with end users who use a system or prototype.
- Articles not providing specific results for older adults aged ≥60 years. Thus, we excluded articles that did not offer results distinguishing between older adults and other user groups, such as caregivers or experts who were not necessarily old. We also excluded articles that did not explicitly match the results within a range of years, for example, articles in which only the average or median age was mentioned.
- Articles exclusively focusing on the contribution to hardware development.
- Full-text articles were inaccessible.
- To validate the eligibility criteria, we peer reviewed 12 articles each. Therefore, we decided to integrate the following exclusion criterion:
  Articles in which the results were not transferable either because there are no recommendations about the design of mobile apps for older adults or because the article only provided usability tests that informed the results of its own app, but no design recommendations could be extracted.

### Data Extraction

The 41 articles that met the eligibility criteria were individually reviewed by 2 authors to extract relevant information to later complete the quality assessment stage. All the collected information was stored in a data extraction spreadsheet.

After individual analyses, 6 consensus meetings were held to discuss the results obtained and reach an agreement on the extraction of qualitative data, such as errors identified through usability testing, design recommendations, or publication venues. In each of the 6 meetings, we peer reviewed 10 to 12 articles and documented the results in a collaborative spreadsheet on Microsoft Teams (Microsoft Corporation). During the analysis, 1 author used the snowballing method, which eventually allowed us to include 5 more articles. The different aspects that were analyzed in each article are presented in Textbox 2.

**Aspects considered during article analyses.**

- *Title and digital object identifier* of the article.
- *First impressions and a summary* of the article to point out the features that were considered important but were not covered in the following fields.
- *Number of older participants* involved in the usability testing. For cases in which >1 group of users participated in the evaluation, we considered only those groups that included users aged ≥60 years. If different iterations of usability testing were performed, we analyzed whether the participants were the same in each iteration. If the participants remained throughout the iterations, all were considered. In contrast, if the participants differed in each iteration, we considered only 1 group (the largest group of participants). This item allowed us to perform the following quality assessment: the higher the number of participants, the more robust the results were considered to be, and therefore, more relevance was assigned to the article.
- Article’s venue and quality based on its relation to human-computer interaction (HCI; assessed considering the aims and scope of the venue) and the position of the venue in quality rankings, using sources such as the Journal Citation Report from Clarivate for journals or the Computing Research & Education ranking of conferences. We considered articles published in an HCI venue to have undergone a stricter methodological scrutiny for the usability testing. This item was added to perform the following quality assessment: the more the venue is related to HCI and the better its quality ranking, the more relevance was assigned to the article.
- *The number of times the participants used the system* was evaluated. “One-cut study” stands for evaluations in which participants used the system only once, as compared with longitudinal studies in which the time was expressed in weeks. Time was relevant to the quality assessment because it reflected the reliability and robustness of the results presented in the article. In other words, a longitudinal study can offer more robust results than a one-cut study; therefore, it would be assessed as more relevant.
- *Year of publication*. If the article had both online and printed versions, the earliest date of publication was considered. We believe that newer results should be better aligned with the current technology and technological abilities of the older population. This is essential because these aspects evolve, and the results become obsolete very quickly.
- *Methodological soundness*. We focused on whether the article provided sufficient information about the design of the evaluation, which included recruitment, eligibility criteria, test protocol and procedure, and participants’ demographics. The existence of this information increases the external validity of the results and affects the relevance of the article in the quality assessment stage.
- *Number of tasks defined in the usability test*. We considered a usability test that prompted the user to perform several tasks to provide more reliable results than a usability test with fewer tasks.
- *Venue of the usability test*. Although this is not a central issue in assessing the articles, this item offered us complementary information on the characteristics of usability tests performed with older adults in the literature.
- *The existence of approval from an ethical committee*. We decided that such approval was not essential to assess the articles because obtaining approval to perform a usability study was not required by all journal venues. However, it provided complementary information about the articles.
- *Design recommendations*. We extracted the explicit design recommendations for mobile app design. Accordingly, we excluded participants’ wishes and preferences, as they were not tested. If the study used mixed methods, we divided the recommendations according to each method. We included the recommendations if they were based on usability tests or a mixture of methods (including a usability test). However, we excluded the recommendations based exclusively on methods other than usability tests as well as recommendations from participants who were not older adults.
- *Errors* in the system identified during usability testing. These were compiled to explain the sources of design recommendations. Errors were extracted according to method used.
- *The level of agreement between reviewers* (0%-100%) and comments on the authors’ discrepancies when reviewing the article. This item was added to register the discussions that the reviewers had during data extraction, as this information may be useful for the subsequent data analysis.

### Quality Assessment

The same 2 authors who performed the data extraction in the previous stage used 3 qualitative values (high, medium, and low) based on some of the data extracted to assess the quality of each article considering 4 dimensions.

#### Dimension 1: Is the Quality of the Venue Sufficient?

If the venue was prestigious in the HCI field, it was rated as high. If the venue was not highly ranked but was related to HCI or if the venue was highly ranked in other fields but not related to HCI, the venue was rated as medium. Finally, if the venue was not prestigious in any field and was not related to HCI, it was rated as low. For this assessment, we used the Journal Citation Report and Computing Research & Education rankings as well as the authors’ knowledge.

#### Dimension 2: Do the Authors Use and Describe a Proper Methodology for Evaluation in Their Article?

We considered the testing protocol as the most relevant information, rather than recruitment, eligibility criteria, and participants’ demographics. If the methodology contained all the items, the rating was high. If the methodology contained all items except the testing protocol or procedure or if the methodology contained the testing protocol or procedure but not the other items, the rating was medium. Otherwise, the rating for this dimension was low.

#### Dimension 3: Are the Number of Tasks Tested and the Number of Users Involved Sufficient to Obtain Robust and Reliable Knowledge?

We weighted the number of users more than the tasks used because some studies did not necessarily involve tasks in their usability testing; therefore, the number of users was more important to us. If the number of users and tasks were higher than 10 and 5, respectively, the article was rated as high. If 5 to 10 users participated in the evaluation and the number of tasks was ≤5, a medium rating was given. If the number of users and tasks were <5, we rated it as low.

#### Dimension 4: Can We Extract Guidelines From the Usability Tests?

Even though the existence of transferable results was already an exclusion criterion, the authors evaluated the extent of this transferability based on their experience and previous knowledge, considering not only the number of results but also the extent to which the findings could be generalized.

#### Procedure

The results of the quality assessment were then transferred to Parsifal using the following scheme: for each of the 4 questions, we assigned 4.0, 2.0, and 0.0 points if qualitative ratings were high, medium, and low, respectively. The total score was the sum of the 4 question scores. The cutoff total score to discard low-quality articles was ≤2.0 points (all values were low and only 1 medium, at most). Consequently, 6 articles were excluded because of their low quality.

### Thematic Analysis

The 2 authors who were not involved in the data extraction and quality assessment stages performed the data analysis using qualitative thematic analysis. The purpose of this division of labor was to ensure that the data analysis was not biased by the performance of the previous stages.

Our goal was to identify, analyze, and interpret patterns of meaning within a set of design recommendations. We performed 3 rounds of analyses. The first round consisted of grouping guidelines according to the terms’ commonalities. Then, we organized the concrete guidelines into broader recommendations. In the final round, we grouped the recommendations into themes using a holistic approach. The resulting themes and categorization of the guidelines were first analyzed individually by all the authors and then discussed at 3 consensus meetings to agree on the results. We paid special attention to the consistency of the results and clarity of the terms used in the categorization. During this process, we used a shared spreadsheet to organize the data and Miro (Miro Corporation) to visualize the data. We also used a top-down representation of recommendations ranging from general to concrete. On the basis of our individual analysis of the results and discussions during the consensus meetings, we decided to exclude the following recommendations:

1. *Guidelines based on other’s literature*, that is, guidelines that are not tested with users. Some articles pointed out design recommendations that were extracted from literature references and were not directly tested (eg, Pereira et al [35]).
2. *Guidelines that applied to concrete mobile apps and cannot be transferred*. Some articles made recommendations that we considered too specific to be generically applied. For example, specific display measures such as “button sizes should be at least 200 mm ^2^” [36] or banning specific UI elements such as “do not use the picker” [37]. We considered that this type of guideline required further experimentation.
3. *Guidelines that were not sufficiently argued in the article*. Some articles pinpointed recommendations without explanation; therefore, they were excluded. For example, the recommendation “allow the user to select the icons preferred” [38] was not sufficiently argued and contradicted with other guidelines that did not allow such flexibility.
4. *Guidelines that were supported in only 1 article*. If the recommendation appeared in a single article, it was ignored because it was not considered generalizable, for example, “Maintain link underlined” [39].

### Workflow

**Figure 1:**
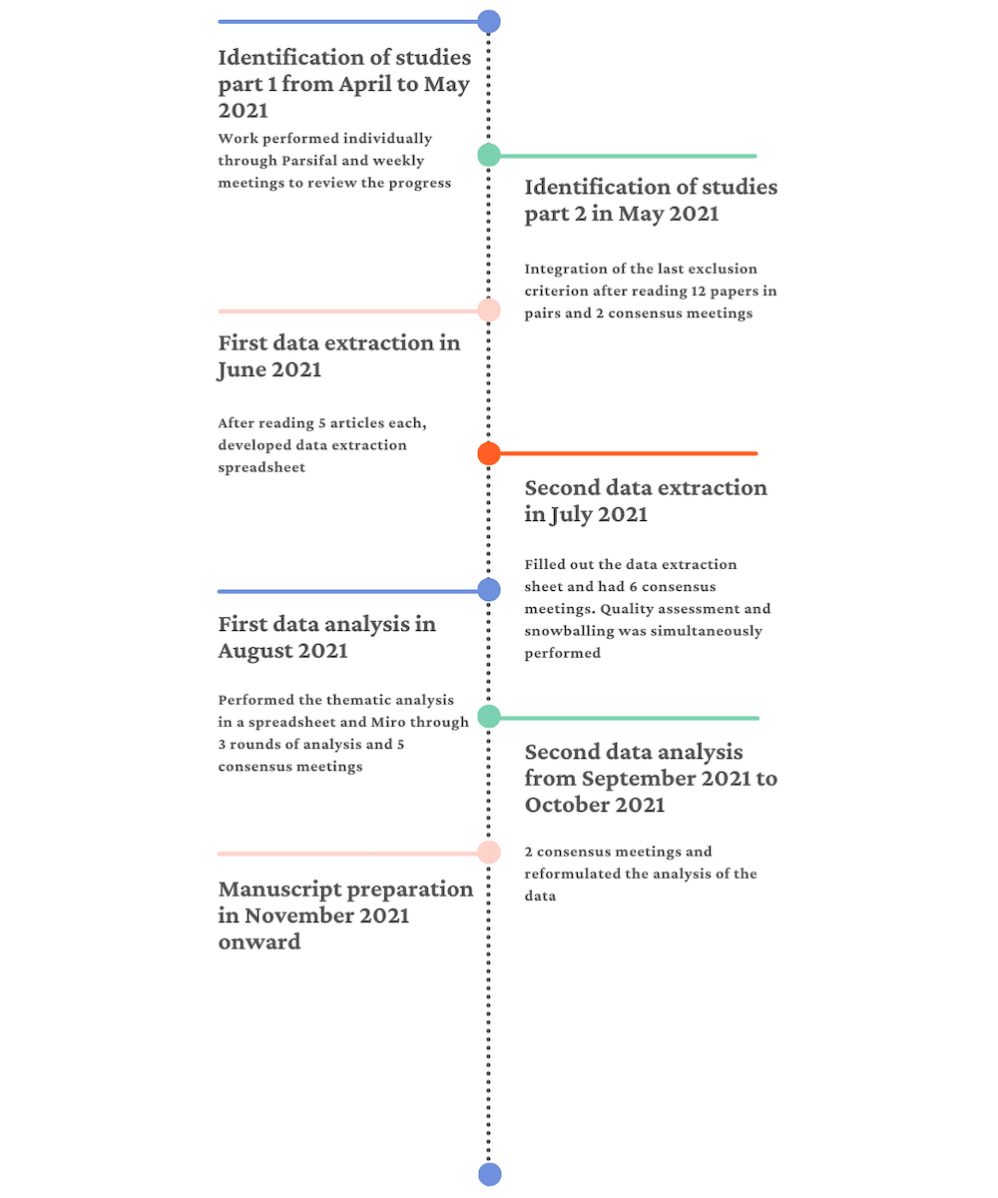
Workflow of the study.

From the search strategy through the thematic analysis stage, we worked individually and held consensus meetings along the way to agree on certain criteria, strategies, tools, and so on. First, we identified the studies; second, we extracted data from the selected articles; and finally, we analyzed the data thematically. We performed the review for 6 months before manuscript preparation. The workflow is illustrated in Figure 1.

## Results

### Systematic Review Results

#### Overview

**Figure 2:**
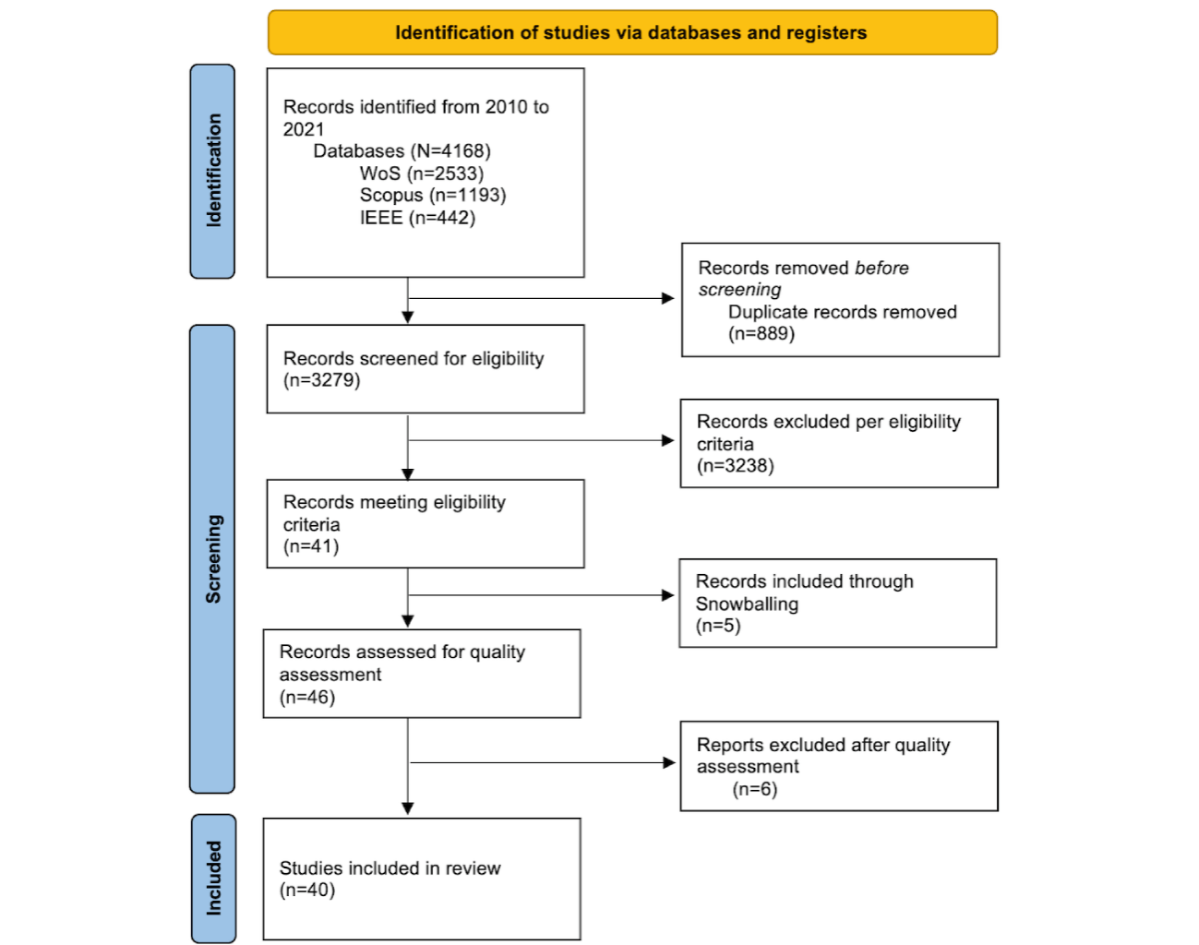
PRISMA (Preferred Reporting Items for Systematic Reviews and Meta-Analyses) flowchart for study selection. WoS: Web of Science.

A total of 40 primary studies were retrieved. The initial search yielded 4168 articles (n=2533, 60.77% from Web of Science; n=1193, 28.62% from Scopus; and n=442, 10.6% from IEEE). Removal of duplicates decreased the number of articles to 3279. Through the screening of abstracts, titles, and keywords, 3238 articles were excluded. Subsequently, we assessed the full text of these articles, resulting in a final number of 40 studies. Figure 2 illustrates the flowchart of the review process.

On the basis of the exclusion criteria, the number of articles excluded (n=3238) for each criterion was as follows: the technologies at play were not mobile (n=343, 10.59%), articles had <4 pages (n=30, 92.65%), the researchers were not able to access the full article (n=1, 0.03%), articles were not written in English (n=6, 18.53%), the methodology did not include a usability test (n=1165, 35.98%), the results could not be transferred (n=94, 2.9%), there were no specific recommendations for older people (n=1583, 48.89%), and articles did not deal with software technology (n=16, 0.49%).

#### Study Characteristics

Of the 40 studies, 16 compared features of mobile touchscreens and were not related to a concrete domain; 17 studies were related to the health care domain; 5 studies addressed social engagement; 1 study concerned entertainment; and 1 was related to housing.

With respect to the number of participants involved in the usability tests, 10 studies involved 3 to 9 participants, 11 studies performed tests with 10 to 19 participants, 14 with 20 to 29 participants, and 5 with >30 participants.

Regarding the duration for which the participants tested the apps, 35 studies encompassed only 1 test session, and 5 investigations were longitudinal. Among the latter, 2 tested the design for ≥1 month and 3 between 1 and 4 weeks. Although the number of tasks used in the usability tests was unspecified in 9 studies, 26 studies performed 1 to 9 tasks, and in 5 studies, 10 tasks were completed. The venue of the test was unspecified in 18 studies, whereas 10 studies performed the usability tests in a laboratory, 4 in day-care centers, and 8 in the participants’ homes. Of the 40 studies, 12 (30%) received ethics approval to perform their tests.

### Thematic Analysis Results: Guidelines

#### Golden Rules

There are 2 guidelines of special significance, as both have been mentioned in 15 studies:

- *Simplify* [36,37,39-51]. There were different recommendations in the 15 studies, but they can be summarized as the need to simplify the design. The cognitive difficulties experienced by these users call for extra effort to simplify the product concept and any element that the users need to understand to successfully operate the mobile app.
- *Increase the size and distance between interactive controls* [36,39,41,43-45,49,52-59]. The size of interactive controls (eg, buttons or form entries) should be augmented to facilitate older people’s interactions with them. Spacing should also be large to avoid accidental tapping because older users with motor limitations may have less precision in interacting with controls. If possible, a touch area that exceeds the visual component should be defined.

**Figure 3:**
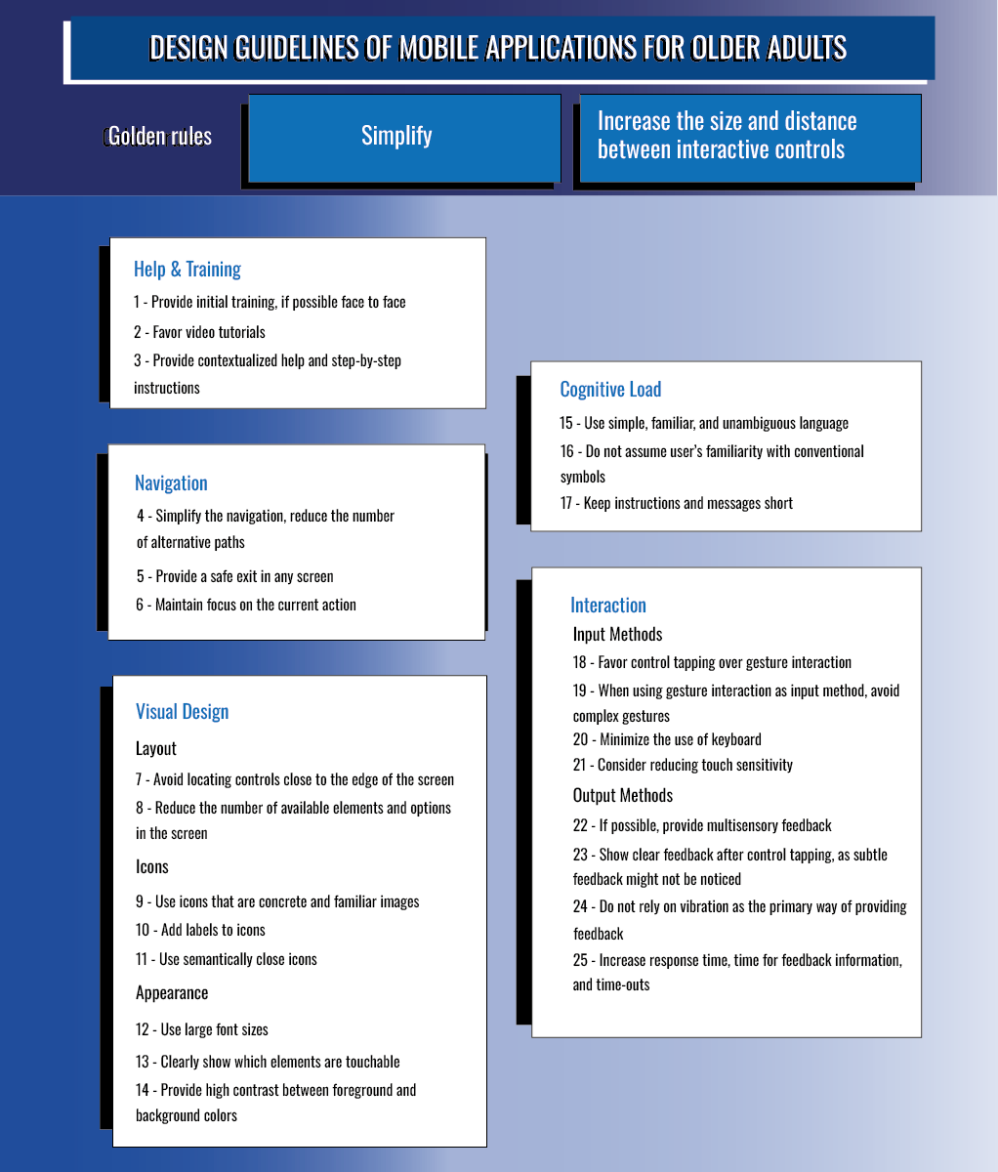
Summary of the guidelines.

Because these 2 recommendations were recurrent and of greater significance than the rest of the guidelines, we have chosen to represent them separately as golden rules that should be followed in the design of any mobile app to be used by older users. The rest of the recommendations gathered have been expressed as guidelines, grouped into 5 categories, as detailed in the following subsections and shown in Figure 3.

#### Help & Training

**Figure 4:**
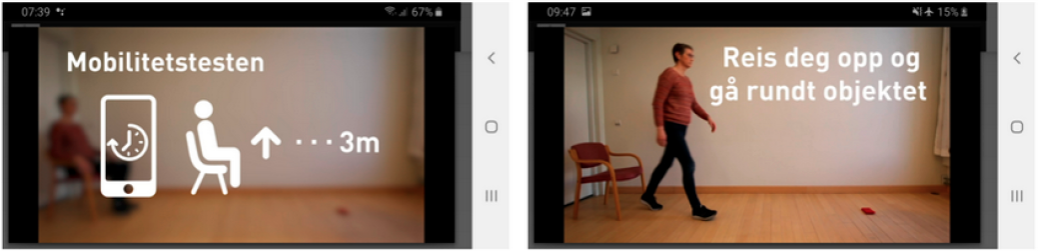
Example application of guideline 2, “Favor video tutorials” (from Bergquist et al [62]).

This category includes guidelines 1-3:

- *Provide initial training, if possible face to face* [52,60]. Considering the difficulty in providing initial training, a face-to-face demonstration of the system before its first use should be provided, as this population requires special support. The lack of familiarity with technology may affect the ability of a significant number of older users to benefit from written documentation. Face-to-face training would be most helpful for critical apps (health, personal finance, etc).
- *Favor video tutorials* [61-63]. When an older person is learning how to use an app, it should offer help through a video rather than written instructions. Written instructions can complement the videos; however, they should not stand alone. For example, Bergquist et al [62] provided instructional videos explaining how to perform the tests included in their app (Figure 4).
- *Provide contextualized help and step-by-step instructions* [41,62,65]. Having to search the help subsystem for how to solve a specific problem can be very time-consuming for older users and sometimes unsuccessful. Guidance on how to use the system, especially for complex tasks, should be structured and accessible. Designers should provide contextual help by focusing on the visible interface context.

#### Navigation

**Figure 5:**
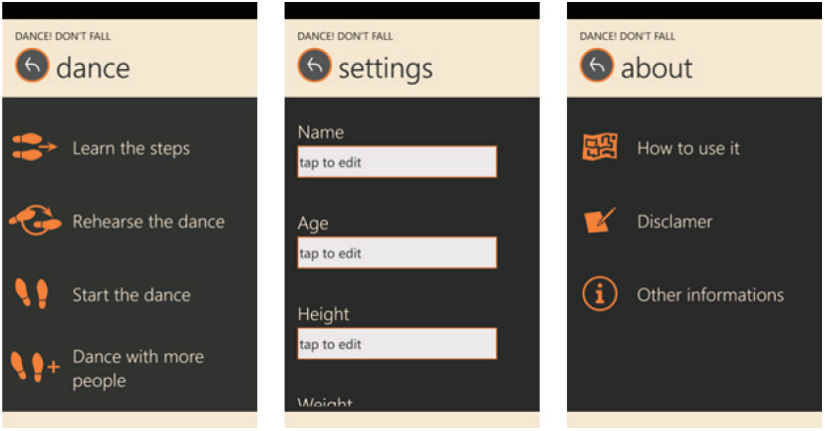
Example application of guideline 5, “Provide a safe exit in any screen” (from Barros et al [53]).

This category includes guidelines 4-6:

- *Simplify the navigation, reduce the number of alternative paths* [40-42]. Navigating through a mobile app can pose a significant challenge for users with cognitive difficulties because they may be lost if they cannot remember all the steps to perform a task. We need to provide a navigation that is simple and uses logic involving very few rules, works everywhere, and reduces the number of alternatives. For simpler navigation, the complexity of the number of optimal paths and optimal path length should be reduced.
- *Provide a safe exit in any screen* [39,53]. Make sure that every screen includes an apparent exit on the interface, so that older adults can avoid anxiety when they do not know what to do in the app. By safe exit, we mean any way to return to a previous safe state, such as a return function; the back button (as in the app designed by Barros et al [53], displayed in Figure 5); or a cancel option.
- *Maintain focus on the current action* [39,66]. Because older users will have more difficulties maintaining concentration, we need to help them focus on the current action. Do not display secondary functions. Instead, attract users’ attention to the most important or typical button to tap in the next step. Thus, older users will be able to proceed more easily through the app and avoid navigation difficulties.

#### Visual Design

##### Layout

This category includes guidelines 7 and 8:

- *Avoid locating controls close to the edge of the screen* [53,67]. Position interactive elements far from the edge of the screen, so that older adults will avoid involuntary interactions when using the mobile app.
- *Reduce the number of available elements and options in the screen* [43,54,64]. Simplify the layout, even at the cost of reducing the set of available functionalities. Older adults tend to have a better UX with an app when layouts are simple.

##### Icons

This category includes guidelines 9-11:

- *Use icons that are concrete and familiar images* [60,68,69]. The use of abstract icons should be avoided, and icons should depict real-world representations. For example, add graphical content to labels such as medication package pictures that are meaningful to older users.
- *Add labels to icons* [39,44,45,68-70]. To improve understandability and user performance with a system, designers should add textual support to icons and buttons. The textual support should represent the purpose of the icon.
- *Use semantically close icons* [45,68,70]. “Semantic distance refers to the closeness of relationship between the icon and the function it represents” [71]. A small semantic distance will have a positive impact on icon recognition by older people.

##### Appearance

This category includes guidelines 12-14:

- *Use large font sizes* [36,41,43,72]. Visual acuity diminishes with age; therefore, a large font size will help older users read the text. Ensure that the font size is sufficiently large to be visible to older people.
- *Clearly show which elements are touchable* [45,66,73]. Users experiencing cognitive strain will have more difficulty distinguishing interactive elements from noninteractive ones. In addition, older users may not be familiar with conventional affordances in mobile apps. Therefore, enhance the difference between touchable and nontouchable elements at the interface through clear boundaries and avoid ambiguous elements. For instance, enable cues for interaction so that the older person knows whether an element is selectable or draggable.
- *Provide high contrast between foreground and background color* [39,41,44,72]. Visual acuity diminishes with age, so a strong contrast between the text color and the background color will help older users read the text. Even if it mostly benefits users with visual impairments, this recommendation can eventually benefit a broad range of users, for example, when using a screen with low brightness or when fatigued.

#### Cognitive Load

**Figure 6:**
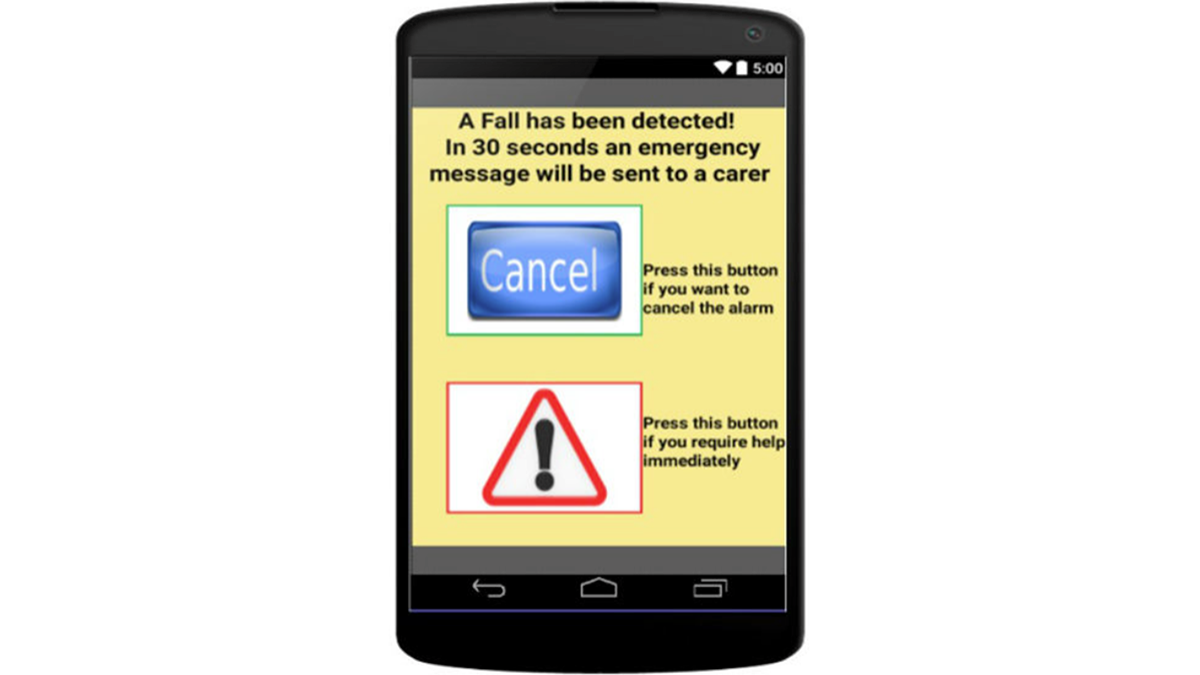
Example application of guideline 15, “Use simple, familiar, and unambiguous language” (from Harte et al [36]).

This category includes guidelines 15-17:

- *Use simple, familiar, and unambiguous language* [36,53]. Users who lack familiarity with technology will find it challenging to interpret technical terms and common symbols used in the UI. They will have added difficulty coping with ambiguous language because they lack the heuristics for interpreting the UIs that technology-proficient users possess. Use simple terms and clear feedback in mobile apps. In this way, older users, regardless of their cultural background, can understand it, and the technology does not cause as much anxiety. Harte et al [36] use an example of simple, familiar, and unambiguous language as shown in Figure 6.
- *Do not assume users’ familiarity with conventional symbols* [44,60,74]. Designers should not take for granted that older users will understand usual conventions, such as “?” being a help button or “→” being a send button. They should create symbols that are understandable and adapt to the cultural context of the person, regardless of their familiarity with technology.
- *Keep instructions and messages short* [37,63]. Instructions and text on how to use a system should be short to avoid overwhelming older users with the cognitive effort of reading extensive messages.

#### Interaction

##### Input Methods

This category includes guidelines 18-21:

- *Favor control tapping over gesture interactions* [47,55,75]. Favor direct manipulation on the screen (control tapping or single tap) over gesture interaction. The latter requires advanced motor skills that may be difficult for older users and can hinder good UX with a system. By control tapping, we mean requiring the user to place the finger over a specific control appearing on the screen, as opposed to making a gesture such as pinch or swipe. For example, Barbosa Neves et al [75] designed an app whose only input interaction was single control tapping.
- *When using gesture interaction as input method, avoid complex gestures* [39,50,76]. We cannot rely on users remembering gestures because there is no hint in the UI that helps the users to recall the set of available gestures. Due to skin aging, wrinkling, or hand tremors, older users may lose contact with the screen, and the gesture may not be correctly interpreted by the system. This adds to the problem of lack of familiarity with technology, as gesture-based interaction is an advanced feature, aside from perhaps a very common gesture such as pinch to zoom.
- *Minimize the use of keyboard* [53,54,73]. Virtual keyboards require fine motor abilities, which are difficult for older users with hand tremors or arthritis. As a possible alternative, the use of voice input could be explored and usability tested.
- *Consider reducing touch sensitivity* [64,74]. A high sensitivity to touch produces involuntary taps on the screen by a certain number of older users. Designers should consider how high control sensitivity is and consider reducing it if there is a risk of involuntary taps by older users. Thus, these users will be able to move their hands over the screen with less fear of accidentally tapping on the controls.

##### Output Methods

This category includes guidelines 22-25:

- *If possible, provide multisensory feedback* [4,39,49,58,60]. Because older users may experience perception limitations, multisensory feedback will increase the probability that messages will get to users correctly. In this manner, we provide multiple options to users who have limitations in hearing or vision.
- *Show clear feedback after control tapping, as subtle feedback might not be noticed* [56,74]. Limitations in perception may lead the user to miss subtle feedback; therefore, feedback should be clear and always provided as a response to an explicit user action, such as control tapping. Older users may not notice subtle changes in the color of a pressed button, and they have a higher risk of tapping outside the target. Therefore, provide bolder interaction feedback anytime the tap has occurred so that the user is aware of having tapped a control.
- *Do not rely on vibration as the primary way of providing feedback* [49,67]. Designers should not consider vibration and tactile feedback as the only means of conveying information because older users may not notice it. Current mobile phones provide weak vibration motors, but this could change in the future.
- *Increase response time, time for feedback information, and time-outs* [4,39,57,61]. Long time-outs in input interaction modes allow users time to interpret the screen and decide on their next action. In this regard, the time for feedback information on the screen should be long enough for users to process, as, for example, in the case of pop-up messages.

## Discussion

### Principal Findings

The guidelines obtained address various issues at diverse abstraction levels. This is due to the method used to obtain the guidelines, as different studies made recommendations at different abstraction levels. We omitted solutions that were applied only to a specific design and were not easily transferrable to other problems and domains, as mentioned in the *Thematic Analysis* section.

Overall, we believe that designers should choose design options that would benefit older users without the need to create a different version of the mobile app. Thus, the same mobile app could be used by a wide range of users, regardless of their age. This approach aligns with the Universal Design perspective, which embraces “the design of products, environments, programmes, and services to be usable by all people, to the greatest extent possible, without the need for adaptation or specialized design” [77]. We acknowledge that it may be necessary to either make adaptations for users with severe limitations or create a simplified mode for older users when complex operations are necessary for other users. However, we believe that it is wrong to disregard older users from the beginning. Designers should adopt the philosophy of trying to choose the design options that can best accommodate older users without losing other users.

The golden rule of *Simplify* is at the highest level of abstraction. Simplicity is related to higher UX. People love designs that make their lives simpler [78], so it is a guideline not just for older users, who not only prefer a simpler system but also require it. Cognitive limitations associated with age increase the challenges of understanding and remembering how to use a complex system. The aim of reducing complexity for older users aligns with the recommendations of Fisher et al [79].

Eyesight and motor limitations in older users necessitate the golden rule of *Increasing the size and distance between interactive controls*. Reduced motor skills cause older users to have more trouble when tapping small controls or controls that are too close together. Web design guidelines for older users, such as those developed by Kurniawan and Zaphiris [80], identified the need to provide larger targets for these users. Touchscreens already require larger touch targets than desktop or web systems accessed with a mouse, because fingers are much bigger than a pointer and have much less precision in target selection compared with mouse clicking [81]. For older users, this problem can be aggravated by their lack of dexterity and motor skills.

The *Help & Training* guidelines provide specific advice on how to provide proactive help [82]. Given that a certain number of older users will not be familiar with technology, learning through exploration is not a good strategy to favor this type of users. The survey by Leung et al [83] confirmed that preference for trial-and-error strategies decreases with age when learning to use mobile apps. The survey also found that older users were more interested in demonstrations followed by an opportunity to replicate steps to obtain feedback, compared with internet information or contact with help-desk staff.

The recommendation to favor video tutorials over written instructions matches our own experience in performing usability tests on eHealth apps for geriatric patients, wherein we observed users tiring easily from lengthy textual instructions [84,85]. Although the study by Ahmad [86] focused on younger tech-proficient adults (aged >50 years), it showed the user preference for video tutorials. In addition, designers should be aware that a significant proportion of the older population has a low literacy level [19]. In our experience, this is especially relevant in usability testing with older users in a medium- to low-income neighborhood [84,85]. Relatedly, Androutsou et al [87] identified the difficulty that older users experience when reading text in notifications. These findings also relate to the guideline that recommends keeping instructions and messages short.

The heuristic on help and documentation by Nielsen [88] advises listing concrete steps to be completed. Older users will better understand a set of instructions if they are provided step-by-step as they proceed with their task with the app. The study by Leung et al [68] showed that older users place more significance on learning task steps than on gaining a general understanding when learning to use mobile apps.

By reducing the number of possible alternatives in the app, we offer less freedom to the user; however, we can reduce the length of the optimal path. For procedural tasks, such as health-monitoring activities (as the ones described by Villalba-Mora et al [84] and Moral et al [85]), it is preferable to use a wizard navigation style in which users are only offered 2 options: go to the next step or exit. Such a simple navigation scheme helps most older users avoid the cognitive burden of deciding where to tap next to continue with their tasks.

The suggestion to maintain focus on the current action follows these same principles. Secondary functions, even if valuable to a minority group of users, can distract a significant number of older users. It is better to attract the attention of the user to the action that we expect to be taken in the most typical scenario, thus preventing user errors [80].

Regarding the need to use larger fonts, Harte et al [36] recommended that for simple interface elements, text sizes should be at least 10 points (Didot system); however, Morey et al [41] recommended using font sizes that are at least 30pts for critical text and at least 20 points for secondary text. Because there is no consensus on the exact minimum font size, we opted to state that the font size should be large to direct the attention of the designer to this issue. This topic needs further research through usability testing.

Reduced motor skills affect the dexterity necessary to properly hold the mobile device and to avoid tapping on an app control on the screen when it is too close to the screen edge. Both golden rules lead to a screen design with less clutter, thereby reducing the number of elements available at a given time. Guideline 8, namely, *Reduce the number of available elements and options in the screen*, explicitly states this corollary to both golden rules. As a result, there is an additional limitation on possible design solutions, which requires extra effort to complete the design work. Every element placed on a screen needs to have a clear purpose and be relevant to most users.

Icons have received substantial attention in the analyzed studies. Although it is a very specific interaction element, icons are relevant in mobile apps and pose a significant difficulty in the design endeavor. According to the study by Leung et al [68], older users identify icon objects and interpret icon meanings less accurately than younger users. The suggestion to use icons that are concrete and familiar images aligns with the recommendation by Petrovčič et al [16] to use meaningful icons, though they refer to the design of mobile phones and operating systems rather than individual mobile apps.

Regarding the recommendation to minimize the use of the keyboard, in the study by Soares Guedes et al [89], almost all study participants (aged 60-76 years) had difficulty using the virtual keyboard because the keys were small and required precision.

In the usability testing of our eHealth apps for older patients, we observed users tapping twice or thrice on the same area of the screen inadvertently when trying to tap just once, due to their lack of soft-movement skills [84,85]. The recommendation to consider decreasing touchscreen sensitivity addresses this problem. Nevertheless, reducing too much sensitivity could lead to the interaction being unnatural for users who are used to a fast response. This topic needs further research by means of usability testing with different types of users.

The effectiveness of vibration feedback for older users remains unclear and open for discussion. Huppert [90] states that in users who are aged >50 years, the ability to perceive vibrations is diminished. Therefore, vibration feedback is not as effective as visual or auditory feedback, supporting the guideline of not using vibration as the primary method of feedback. Because 2 of the studies considered in our review (de Almeida et al [39] and Leitão and Silva [58]) recommended using vibration as one possible multisensory feedback, we have not ruled out its use as such. Nevertheless, further research on this topic is needed.

Huppert [90] also identified the age-related decline in the speed at which information is processed as the reason why older users have difficulties with tasks in which information is presented for very brief periods or rapid responses are required. Therefore, the last of the compiled set of recommended guidelines is to *Increase response time, time for feedback information, and time-outs*.

### Limitations

One limitation of this study is that potential errors could have occurred during the revision and synthesis of the articles. To avoid this, we performed these tasks in pairs and held periodic meetings along the way. Two reviewers extracted the data, and 2 different authors performed the thematic analysis. The screening was performed by 1 author, because reviewing 4174 articles was best done alone rather than in pairs. Furthermore, to prevent errors, we performed the snowballing review after the screening to include additional articles that matched our inclusion criteria.

Regarding the age group, we set the minimum age cutoff at 60 years. Different choices for the user group age range could lead to different results. Considering a younger age would include additional studies whose user characteristics differ from those of the target users. By contrast, a higher minimum age cutoff could severely restrict the number of studies included, thus affecting the quality and practical applicability of the results.

Moreover, we extracted studies that performed usability tests and excluded studies that used other methods, such as qualitative inquiries, focus groups, and ethnographic observations. We decided to follow this strategy not only to narrow down the search but also because usability tests are the gold standard for assessing usability. HCI remains our theoretical tenet. Nevertheless, we acknowledge that other valuable guidelines could be proposed in studies that use different methods.

We excluded gray literature and unpublished articles, so the sources were limited to articles in journals and conferences with data that have gone through a thorough review process. Due to the considerable number of articles screened, we believed that the number of articles to review was sufficient. We used 3 databases that could have limited our screening phase; however, we believe that Web of Science, Scopus, and IEEE are the broadest and most relevant to the HCI field. Moreover, we trialed preliminary keywords in different databases because the search strategy could have missed relevant articles.

### Conclusions

There is sufficient experimental evidence in the published literature about design features in mobile apps that improve usability for older adults. We systemically extracted design recommendations stemming from this experimental evidence, where usability tests were conducted with actual older users. Through a thematic analysis, we organized these findings into a set of 27 recommendations, including 2 golden rules and 25 design guidelines classified into 5 categories (Help & Training, Navigation, Visual Design, Cognitive Load, and Interaction).

Design guidelines do not ensure usability or a good UX on their own, but we hope that these design guidelines will support the design of better mobile apps through a user-centered design approach, catering to the needs and characteristics of older users and helping bridge the age-related digital divide.

We plan to apply these guidelines to real mobile development projects in which older users are either part of or the entire user target to assess their applicability. Further research is also necessary to gain a deeper understanding of how the different guidelines are nuanced based on the diverse characteristics of older users in terms of age, previous experience with mobile technologies, or physical and cognitive limitations, among others.

## Appendix

Multimedia Appendix 1 PRISMA checklist.

## Abbreviations

HCI: human-computer interaction
mHealth: mobile health
PRISMA: Preferred Reporting Items for Systematic Reviews and Meta-Analyses
UI: user interface
UX: user experience
